# Proteomics Studies on Extracellular Vesicles Derived from Glioblastoma: Where Do We Stand?

**DOI:** 10.3390/ijms25189778

**Published:** 2024-09-10

**Authors:** Patricia Giuliani, Chiara De Simone, Giorgia Febo, Alessia Bellasame, Nicola Tupone, Vimal Di Virglio, Fabrizio di Giuseppe, Renata Ciccarelli, Patrizia Di Iorio, Stefania Angelucci

**Affiliations:** 1 Department of Medical, Oral and Biotechnological Sciences, ‘G. D’Annunzio’ University of Chieti-Pescara, Via Vestini 31, 66100 Chieti, Italy; patricia.giuliani@unich.it (P.G.); chiara.desimone@unich.it (C.D.S.); giorgia.febo@libero.it (G.F.); alessia.bellasame@gmail.com (A.B.); patrizia.diiorio@unich.it (P.D.I.); 2 Center for Advanced Studies and Technology (CAST), ‘G. D’Annunzio’ University of Chieti-Pescara, Via L Polacchi 13, 66100 Chieti, Italy; nicola.tupone@studenti.unich.it (N.T.); dvgiampi7@alice.it (V.D.V.); fabrizio.digiuseppe@unich.it (F.d.G.); 3 Department of Innovative Technologies in Medicine and Dentistry, ‘G. D’Annunzio’ University of Chieti-Pescara, Via Vestini 31, 66100 Chieti, Italy; stefania.angelucci@unich.it; 4 Stem TeCh Group, Via L Polacchi 13, 66100 Chieti, Italy

**Keywords:** glioblastoma multiforme (GBM), glioblastoma-derived stem cells (GSCs), extracellular vesicles (EVs), proteomic studies, GBM biology, GBM biomarkers, discovery of druggable targets

## Abstract

Like most tumors, glioblastoma multiforme (GBM), the deadliest brain tumor in human adulthood, releases extracellular vesicles (EVs). Their content, reflecting that of the tumor of origin, can be donated to nearby and distant cells which, by acquiring it, become more aggressive. Therefore, the study of EV-transported molecules has become very important. Particular attention has been paid to EV proteins to uncover new GBM biomarkers and potential druggable targets. Proteomic studies have mainly been performed by “bottom-up” mass spectrometry (MS) analysis of EVs isolated by different procedures from conditioned media of cultured GBM cells and biological fluids from GBM patients. Although a great number of dysregulated proteins have been identified, the translation of these findings into clinics remains elusive, probably due to multiple factors, including the lack of standardized procedures for isolation/characterization of EVs and analysis of their proteome. Thus, it is time to change research strategies by adopting, in addition to harmonized EV selection techniques, different MS methods aimed at identifying selected tumoral protein mutations and/or isoforms due to post-translational modifications, which more deeply influence the tumor behavior. Hopefully, these data integrated with those from other “omics” disciplines will lead to the discovery of druggable pathways for novel GBM therapies.

## 1. Introduction

Glioblastoma multiforme (GBM), usually indicated as glioblastoma, is the most frequent and severe form of glioma that affects the human central nervous system in adulthood. It can be diagnosed as primary (de novo formed) and secondary (arising from lower grade tumors) GBM, the former being more aggressive and with a poorer prognosis for patients than the latter. Primary GBM, indeed, shows intra- and inter-tumoral heterogeneity, ascribed to multiple mutations occurring in different patients. Despite aggressive treatments, comprising neurosurgery, radiotherapy, and chemotherapy, GBMs show a persistent growth, which is primarily ascribed to the presence of a perivascular niche within the tumor mass containing stem-like cells, namely glioblastoma stem cells (GSCs). These cells, like others in different tumors, are provided with a high proliferative rate, differentiation ability in various tumor cell types, and resistance to the current therapeutic interventions [1]. They may originate from the subventricular zone (SVZ), from which they spread toward the frontotemporal cortex and lobe, where they gradually accumulate mutations giving rise to primary GBM. However, a secondary hypothesis attributes the origin of primary GBMs to outer radial glial progenitors deriving from astrocytes with high expression of a tyrosine kinase receptor ErbB2, which supports neural cell proliferation and motility [2]. Last but not least in GBM growth and resistance is the role played by the tumor microenvironment (TME) [3], which supports the tumor survival and expansion, favoring close communications between GBM cells and surrounding non-tumor cells. These occur through direct cell-to-cell contacts and by the mediation of both soluble factors (cytokines, chemokines, and growth factors) and molecules contained in extracellular vesicles (EVs) [4,5].

The existence of these nanoparticles, which are released from virtually all cell types in the extracellular fluids, has attracted great interest in all medical areas, leading to the discovery of their pivotal role in intercellular communications [6]. Indeed, EVs transport a heterogeneous cargo comprising nucleic acids, proteins, and lipids, which can be donated to nearby or distant cells. In this way, EVs can promote angiogenesis, cell migration and invasiveness, immune suppression, and drug resistance in tumors. All these activities are also involved in GBM aggressiveness and recurrence. In their favor, EVs also show great accessibility in biofluids and are therefore proving to be very useful for tumor diagnosis and prognosis, being promising as possible therapeutic tools [7,8].

**Figure 1:**
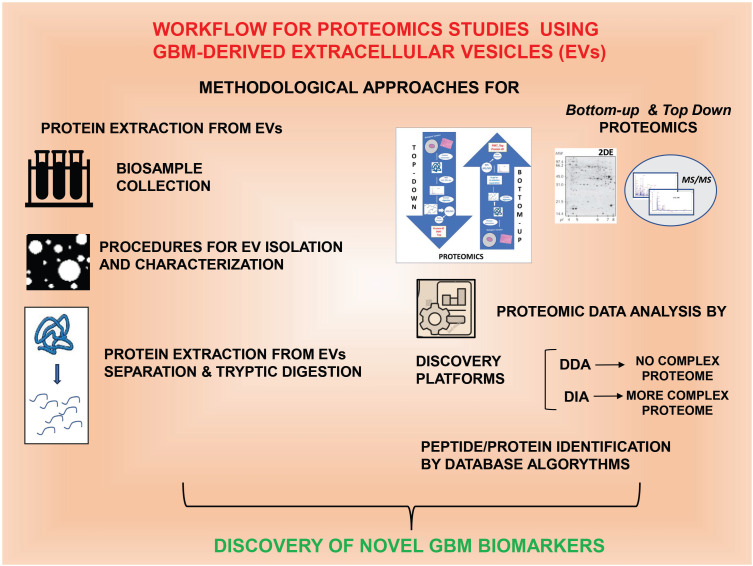
Most used experimental approaches in proteomic studies using EVs obtained from GBM-related biological samples following different procedures. All steps are described in detail in the review text.

In this review, besides briefly highlighting the principal methods used to isolate and characterize EVs, we focused on the studies performed on the proteome of GBM-derived EVs, the importance of which will be discussed in the following chapters (see Figure 1).

## 2. Types of EVs and Methods to Isolate and Identify Them

**Figure 2:**
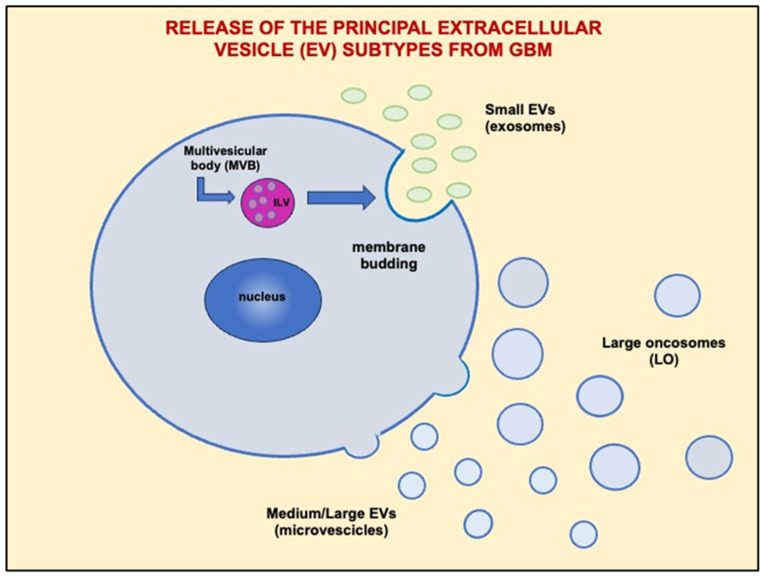
Exosomes, now preferably indicated as small EVs (sEVs) (diameter of 30–100 nm), form from invagination of the endosomal membrane when intraluminal vesicles (ILVs) are secreted during fusion of multivesicular bodies (MVBs) to the cell surface. Microvesicles, now indicated as medium/large EVs (m/lEVs) (diameter of 100–1000 nm), form via an outward budding of the plasma membrane, leading to the release of these vesicles into the extracellular space. Large oncosomes (LO) are larger (1–10 μm diameter) cancer-derived EVs originating from membrane bleb shedding and are associated with disease progression.

Three main types of EVs, namely exosomes (EXOs), microvesicles (MVs), and large oncosomes (LOs), have so far been identified in different cell contexts, including GBM [9,10] (Figure 2). There is also a fourth type of EV, namely apoptotic bodies (1–5 μm size), which are generated during the process of cell death by apoptosis [11]. Typically, EXOs are the smallest EVs, with a size ranging from 30 to 100 nm. They derive from the plasma membrane budding inward to form multivesicular bodies (MVBs), from which intraluminal vesicles (ILVs) are generated and, after fusion with plasma membranes, are released in the extracellular space as EXOs. MVs have a larger size (100–1000 nm) and derive from outward blebbing of the plasma membrane through a complex process influenced by the cell’s physiological condition and microenvironment. LOs, more recently discovered and therefore not yet well characterized, are the largest EVs (1–10 μm) [12]. They are released by tumor cells, including glioma cells [13]. Although the terms and the distinction among EV subtypes reported above are frequently used in the current literature, the adoption of the generic acronym “EVs” has recently been recommended by the International Society for Extracellular Vesicles (ISEV) to indicate all nanoparticles, while the distinction of EV subtypes based on size, density, molecular composition, or cellular origin should be approached with caution. Again, the terms “exosomes” and “microvesicles” are discouraged, replaced by “small EVs” (sEVs) and “medium/large EVs” (mEVs or lMVs), respectively [14].

The isolation of EVs from either GBM samples and glioma cell cultures or, more recently, biological fluids of GBM patients has represented an important step forward for tumor diagnosis and management. So far, glioma classification has mainly been based on tissue biopsy coupled to histological and immunohistochemical techniques, while clinical diagnosis is classically performed through different imaging techniques [15,16]. However, the methods applied to biopsy tissues do not monitor continuous tumor changes, whereas imaging does not reliably distinguish between tumor growth and treatment-induced lesions. In this context, the discovery of EVs has promoted the study and adoption of more advanced and sophisticated models/methods to detect novel tumoral targets to be validated as biomarkers. All this information has contributed to better identifying tumor type as well as potential candidates to hopefully develop new therapeutic strategies [17,18].

The isolation and the distinction between tumor and nontumor-derived EVs are challenging and represent the first crucial point in studying EV functions [19]. Most research on this aspect, mainly in the past, has been performed on a conditioned medium in which different glioma cell lines were grown. New important data have more recently been obtained by liquid biopsy, which is a non-invasive technique that can be applied to body fluids such as cerebrospinal fluid (CSF), blood, urine, and, more recently, also saliva, therefore being especially useful in the clinic [20,21,22,23]. However, contaminant proteins are present both in cell culture media supplemented with serum and in patients’ biological fluids, which can affect the EV isolation and purification procedures. The protein concentration is particularly elevated in these fluids and proteins cover the EV surface like a “corona”. Of note, these proteins are not always simply contaminants but can be also coupled to EV functions [24,25].

Therefore, it is conceivable that the procedures to isolate EVs are multiple, all techniques aiming at the enrichment and purification of the nanoparticle fraction from the original source (Table 1).

When using tumoral tissue to isolate EVs, tissue biopsies should be immediately fresh frozen at −80 °C and, at the time of use, small tumor tissue pieces must be treated enzymatically and homogenized to obtain a cell suspension. The latter, as well as any biological fluid (i.e., culture medium or fluid obtained from GBM patients), are subsequently processed by different conventional techniques to obtain sufficiently purified EVs. The principal methods include (i) differential ultracentrifugation (UC) or density gradient ultracentrifugation (DG-UC) of the investigated samples, (ii) EV size-based isolation by ultrafiltration (UF) procedures or size exclusion (SE) chromatography, and (iii) commercial kits using separation technology based on EV–polymer interaction and precipitation or immunoaffinity capture due to antigen-antibody binding. Despite the numerous procedures so far adopted, each of the methods listed above presents some disadvantages (see Table 1), as also highlighted in a recent review, which results in variability in the yield and purity of EV fractions [29]. 

More recent technologies for EV isolation seem to be more reliable. Some of these are based on microfluidics, which is the science of high-precision control and manipulation of fluids that are geometrically forced into networks of small channels, normally less than 100 µm in diameter [30]. This technique has been applied to microdevices and has allowed reduction in the sample volume to be analyzed and the time necessary for the EV isolation process. There is a variety of microfluidic devices using different methods to obtain purified EVs, namely physical or chemical methods. The physical methods can use a passive or active approach. The former isolate EVs by filtering the initial sample through membranes included in microfluidic channels or by exploiting the elastic force of the EVs or of the medium in which they are present. The active approaches exploit physical forces (acoustic waves, electric fields) applied to fluids containing suspended EVs, thus modifying their movement based on their size and allowing the separation of smaller vesicles from larger ones, as well as debris removal. The chemical methods, which are considered the most promising, are based on the chemical affinity between specific antibodies and antigens, thus allowing a more selective recovery of EVs. Obviously, specific substrates such as micrometric solid beads or nanoparticles are required for this method to work (for more detailed information see references [33,34]). Thus, biosensors using aptamers (synthetic nucleic acid oligomers that bind molecular targets, including proteins, with high affinity and specificity) have been developed on various platforms, such as electrochemical, localized surface plasmon resonance (LSPR), surface plasmon resonance (SPR), enzyme-linked aptamer-antibody sandwich (ELAAS), optical, and colorimetric-based platforms [35]. Interestingly, microfluidic platforms can be integrated with optical and spectroscopic devices, among which Raman spectroscopy is one of the most useful [36]. Indeed, a microfluidic device combining nanostructured microchips and micro-Raman technology has been recently applied for GBM-derived EV separation from non-cancerous glial EVs [31,32]. 

According to the most recent position paper published by the ISEV [36], EVs, once isolated from the original source, must be characterized by different methods to ascertain their size, concentration, and purity, as well as the presence of biomarkers such as annexins, tetraspanins (i.e., CD63, CD81, CD9), basigin (BSG), or other transmembrane proteins like SLC3A2 (solute carrier family 3 member 4), which should be selected according to EV source and type [37]. EV characterization also includes their visualization, which can be achieved by atomic force microscopy and electron microscopy. These methods (summarized in Table 2) allow detecting particle shape and morphology, and also evaluating the effects of the isolation methods on their structure [38].

## 3. Proteomics Applied to EVs from Various Sources

### 3.1. Techniques Used to Study the Proteomic Cargo of GBM-Derived EVs

After their characterization, EVs can be used to determine their content, uncover the type of GBM from which they derive, and reveal any other information that may be useful for tumor management. A great help in this direction has been derived from the advent and use of the new sciences globally indicated as “omics”, which include genomics, transcriptomics, epigenomics, proteomics, and metabolomics. This set of techniques have allowed gaining new insights into the biology of GBM. 

Of note, genomics was applied for the first time to GBM, in a project launched by The Cancer Genome Atlas (TCGA), more than fifteen years ago [50]. In this study, several genetic alterations related to *EGFR*, *TP53*, *NF1*, *P13KCA/PIK3R1*, *PTEN*, *PDGFRA*, and *IDH1* were identified in GBM, which were subsequently used to classify GBM tumors into the four genetically defined subtypes, namely classic, neural, pro-neural, and mesenchymal, with the last being the worst (reviewed in [51]). Furthermore, while the neural classification has been removed, further advances in glioma genetic characterization have allowed updating GBM classification as isocitrate dehydrogenase (IDH)-wild type or IDH-mutant, which roughly correspond to primary and secondary GBM, respectively [52]. 

Although the identification of the genetic alterations in GBM has been useful in characterizing the different tumor subtypes and also promoted the experimental introduction of new therapeutic strategies, the prognosis and overall patient survival have not been significantly improved. This is probably due to the great genetic variability of this tumor. In addition, gene transcription does not necessarily correlate with protein abundance [53]. Thus, studies have been enlarged to evaluate the proteomic changes in this tumor, which have assumed a growing relevance, as proteins undergo several post-translational rearrangements that influence the tridimensional structure of the proteins and their activity, and that are dependent also on the microenvironment in which the proteins act. This information is not obtainable from the study of the genome and related RNAs (studied by transcriptomics), while proteomics could do this. It is also important to emphasize that proteins show a greater stability than oligonucleotides, mainly those forming RNAs [54]. Hence, proteomics, which allows the detection of the protein heritage of GBMs, as well as of EVs, directly released from the original tumor, can be used to uncover more cancer targets than genomics [55]. 

Traditional methods for protein separation from biological samples include two-dimensional gel electrophoresis (2-DE) and two-dimensional difference gel electrophoresis (2D-DIGE). Protein spots identified in 2D gels must be digested by proteases to generate peptides for mass spectrometry (MS) analysis. More recently, new techniques such as gas chromatography (GC) or liquid chromatography (LC) coupled to MS have been adopted, which enable simultaneous separation and identification of proteins. Thus, MS has become the most valuable tool in the study of tumors in general and obviously also of GBM, especially for the identification of new potential biomarkers [56,57]. This technique, indeed, exhibits high sensitivity, coupled to the ability to simultaneously identify many proteins. Moreover, it can be applied to different biological sources, both cells in culture and derived from tissue biopsies and biological fluids, such as cell culture medium, TME, and all the body fluids mentioned above, also including those collected by the ultrasound aspirators used during tumor removal [57,58]. Recently, it has been shown that conventional LC and MS can be combined with another technique, ion mobility spectrometry (IMS), which is able to separate gas phase ions based on their shape and size. Advances in this technique have led to the introduction of trapped ion mobility spectrometry (TIMS), which is more versatile than previous IMS instruments, being able to analyze a wide ion mobility range in a few milliseconds (100 ms). Furthermore, TIMS has been coupled to TOF mass analysis, thus increasing the technical performance and resolution characteristics of MS technology [59,60]. Additionally, a MS scan mode has been introduced, namely parallel accumulation serial fragmentation (PASEF), that is able to synchronize TIMS with the MS/MS selection of ion precursors, increasing by more than tenfold the MS/MS acquisition rate, without decreasing the device sensitivity [61]. 

Given the abundance of proteins in tumor samples, methods have been developed to analyze data obtained by MS, such as data-dependent acquisition (DDA) and, more recently, data-independent acquisition (DIA). The former takes into consideration peaks obtained in the first stage of tandem MS from peptides, with ions selected based on their intensity and abundance for fragmentation in a second step of tandem MS. This method analyzes a restricted number of peptides and can generate incomplete or biased data. The latter shows a greater sensitivity and reproducibility in comparison with the former. Indeed, all ionized compounds (mass-range around 25 Da) are fragmented, thus undergoing MS/MS [62,63]. This analysis enables drawing accurate chromatograms for each peptide, even for those with lower expression, allowing their quantitation. A recent comparison between the two analytical methods showed the superiority of DIA in EV proteome analysis and quantification using a very low quantity of nanoparticles (0.5–1 μg) [64]. Data thus obtained, including information on the peptide precursor ion *m*/*z*, the *m*/*z* of the fragment ions with their intensities, and the chromatographic retention time of examined peptides, can generate complex spectra, mainly if data acquisition is performed by full-scan DIA. To reduce the DIA spectra complexity, windowed DIA has been adopted, based on a variable-window isolation strategy to ensure a similar number of precursors are isolated for each DIA fragmentation window across a defined precursor mass range, while mitigating potential chemical and electrical noises [65]. Once obtained, the DIA data analysis must be aligned using an appropriate library containing MS coordinates for target peptides [66]. Given the complexity of DIA-generated spectra and multiplexed chromatograms, informatic tools for data from DIA analysis have been widely implemented, as recently reviewed [65]. 

Findings using this approach are currently indicated as “bottom-up” or “shotgun” DIA-MS data that, summarizing, rely on native proteome digestion, separation of the obtained peptides by chromatographic methods followed by their identification by MS technology, and computerized data analysis using specialized software and databases [67]. Together, this procedure provides a proteome profile, at the same time allowing the identification of novel biomarkers. In this context, it should be added that both DDA and DIA have been combined with parallel accumulation–serial fragmentation (PASEF©) technology, which allows attaining greater sensitivity for quantitative proteomics [59]. 

The literature on the altered protein expression in different GBM samples, such as GBM-derived cells or fluids collected from GBM patients, has become very broad, especially in the last decade, in the hope of identifying possible GBM biomarkers/druggable targets [56]. In contrast, studies on EV proteomics are still poor. However, EVs reflect the protein pattern of cells from which they derive and are able to donate their content to neighboring cells, making them more aggressive. Therefore, the investigation of EV protein content could add important information on GBM biology, which is also useful for tumor management. Here, we performed a review of the literature available on this aspect, also pointing out the critical points that need to be ameliorated to obtain valuable indications from data on the EV proteome.

### 3.2. Proteomics Applied to EVs Obtained from Cultured Cells

Articles investigating the EV protein cargo have used various techniques, depending on the target of the study. Indeed, in the case of papers aimed at the identification/characterization of EV surface markers and/or potential GBM biomarkers useful for understanding tumor response to therapy, the principal techniques used have been Western blot (WB) analysis and/or other immune-based assays (i.e., cytofluorimetry, ELISA assays) [68,69,70,71]. These techniques, however, have also been used to validate the identification of protein sequences from GBM-derived EVs by classic proteomic techniques. 

Many studies have been performed on EVs isolated from the culture medium of commercially available GBM cell lines, which are the reference biological source. Graner et al. [72] were among the first researchers to start investigation on EVs isolated by DG-UC from neural stem cell medium in which murine and human cell lines were grown, as well as from the sera of high-grade glioma patients. Proteins were separated by 2DE and 2D DIGE, while protein spots were analyzed by tandem MS [72]. In brain tumor EVs (characterized as exosomes), the authors selected forty-five proteins, of which 36 had recognized functions in the literature. Besides some common proteins like chaperones (heat shock cognate 71 kDa protein, HSC70, and heat shock protein 60, HSP60), previously identified by WB analysis, surface antigen EGFRvIII and cytokine transforming growth factor beta (TGFβ), proteins with a profile unique were detected in the isolated EVs, such as ARRDC2 (arrestin domain-containing protein 2), 3DPGH (D3-phosphoglycerate dehydrogenase), CRMP2 (collapsing response mediator protein 2), CENP-P (centromere protein P), sialic acid synthase, 3eIF3β (Eukaryotic Translation Initiation Factor subunit 2), proliferating cell nuclear antigen (PCNA), MTAPase (MeSAdo phosphorylase), EF-1β (elongation factor 1β), and PSMA2 (proteasome subunit α type2-C). Furthermore, murine retroviral Gag polyproteins were also identified, which could be related to a retrovirus involved in the progression of murine tumors, as a consequence of the inhibition of immune surveillance. Later on, Shao and colleagues [73] isolated EVs, defined as MVs, from the conditioned medium of GBM cell lines and blood samples of GBM patients. After EV isolation by differential centrifugation and initial characterization of size and concentration by nanoparticle tracking analysis (NTA), the vesicles were analyzed for their protein content. For further purification, blood-derived EVs (again identified as MVs) were introduced in a microfluidic chip, in which MVs were rendered paramagnetic by the use of magnetic nanoparticles targeting their markers. Then, MVs were detected using a miniaturized nuclear magnetic resonance system. For seven specific MV proteins, namely epidermal growth factor receptor (EGFR), platelet-derived growth factor receptor (PDGFRα), podoplanin (PDPN), ephrin type-A receptor 2 (EphA2), EGFRvIII, cytosolic isocitrate dehydrogenase 1 mutation (IDH1 R132H), and cytosolic heat shock protein 90 (HSP90), the identification was performed by FACS and WB analyses. 

A seminal paper in this field was more recently provided by Mallawaaratchy and colleagues [74], which dealt with a wide proteomic profiling of GBM-derived EVs. The study was mostly performed on EVs isolated from six different GBM cell lines, although the findings were compared with those obtained from EVs obtained from fluids collected from human brain tumors (GBM and low-grade glioma) during surgery through a cavitron ultrasonic surgical aspirator (CUSA). The authors emphasized that, from an initial number of 844 proteins in all examined GBM-derived EVs, they identified 145 proteins that were common to the EVs secreted by all cell lines. In particular, they highlighted the presence of 14 proteins related to the formation of invadopodia, which are involved in EV secretion and could be associated with cell invasiveness and a more aggressive GBM type. These data were corroborated by an in silico analysis performed in GBM and normal brain tissue specimens on the expression levels of genes corresponding to the 14 identified proteins. This investigation revealed significantly higher levels of the expression of those genes (especially in classical and mesenchymal tumor subtypes) as compared to normal nervous tissue. Of note, in a more recent article in which different human glioma cell lines were studied, proteomic analysis of their content not only identified cytoskeletal, transmembrane, and GTPase-coupled proteins, but also confirmed the abundance of proteins modulating the formation and proteolytic activity of invadopodia. Additionally, they demonstrated that the secretion of these factors from EVs, as well as their uptake by surrounding cells, was increased by GBM cell treatment with temozolomide and radiation [75].

A different experimental approach was carried out by Choi and colleagues [76], who used the glioma cell line U373, in which the expression of the oncogenic EGFRvIII factor was induced. From the culture medium of these cells, EVs were isolated by DG-UC and the proteomic analysis of their content showed remarkable changes in comparison to the proteome of EVs from control cells (not expressing EGFRvIII protein). In particular, in EVs from U373vIII cells, which were identified as exosomes, there was an upregulated expression of certain tetraspanins (CD151, TSPAN8) that favor intercellular communications mediated by exosomes, while there was a downregulation of certain other proteins, CD81 and CD82, which contribute to the identification and biogenesis of exosomes, respectively. Moreover, the same EVs exhibited high levels of proteases, ECM-related, and cell adhesion proteins, all able to enhance cell invasiveness. Noteworthy, EGFRvIII-transformed glioma cells showed an increased ability to uptake U373vIII-derived EVs, which means that the oncogenic transformation caused by EGFRvIII overexpression in U373 cells modified the properties of both EVs and recipient cells.

Another study by Naryzhny et al. [77] was performed on five different glioma cell lines, from which EVs, again characterized as exosomes, were isolated. In their proteome, a very large number of proteins (133) were identified and grouped according to their function. One of these clusters included several cytoskeleton proteins and chaperones regulating the vesicle assembly, movement, and fusion with membranes. There was also the presence of proteins interacting with nucleic acids, the fragments of which, through the intervention of macrophages and lymphocytes, could participate in the immunological tolerance of tumor cells. Other identified proteins in these EVs were similar to those reported in the proteome of EVs in previous studies, including cell adhesion proteins such as integrins, annexins, tetraspanins (including exosome markers CD9 and CD81), proteases, and related inhibitors. Together, these proteins could promote tumor cell invasiveness, angiogenesis, and immune response modulation. Additionally, further upregulated proteins were identified as involved in glucose metabolism, which could support tumor cell survival or tumor cell resistance to radiation or drugs. The authors, also based on their previous studies, concluded that some of the proteins identified in the exosomes, namely, annexins A1 and A2 (ANXA1, ANXA2), alpha-enolase (ENOA), a member of the glyceraldehyde-3-phosphate dehydrogenase protein family (G3P), heat shock protein 90ß (HS90B), a pyruvate kinase (KPYM), peroxiredoxin 1 (PRDX1), triosephosphate isomerase (TPIS), a member of the AAA ATPase family of proteins (TERA), vimentin (VIME), 14-3-3- protein epsilon (1433E), cofilin (COF1), and nucleophosmin (NPM) could be considered as “GBM biomarkers”, together with CD44 and Tenascin C. However, the authors emphasized that even their list, obtained through MS studies, needed to be implemented using more sensitive methods such as immunological techniques.

Even our group isolated and characterized two subtypes of EVs, at that time indicated as EXOS and MVs, as approved by the ISEV in 2018 [78], the characterization of which was mainly based on morphological details obtained by electron microscopy (TEM) and the expression of selected biomarkers (Alix, CD63, and EPCAM). Of note, differently from all previous studies, which mainly used glioma cell lines, the source of our EVs was GSCs isolated from surgical brain specimens of two patients with primary GBM. EVs were obtained from the culture medium of these GSCs by sequential ultracentrifugation [79], and their proteome was characterized by 2DE analysis combined with MALDI TOF MS/MS. The two EV subtypes, besides numerous proteins in common (more than 1000), showed a conspicuous number of specific proteins with statistically significant expression levels and intensity values. Of these, only those with the most relevant dysregulation were identified by MS analysis. Thus, 21 proteins were selected in MVs and 9 in exosome EXOs. MV proteins mainly included chaperones belonging to heat shock protein 70 (HSP70), which had previously been found in EVs from glioma cell lines [76,80]. These proteins formed an intense network with others including mitochondrial protein GPR75 (stress 70 protein, mitochondrial also known as mortalin), FKBP4 and FKBP5 (peptidyl-prolyn cis-trans isomerases 4 and 5), and Lamin A/C (prelamin-A/C), which have become regarded as indexes of GBM progression. Additionally, the MVs also contained metabolic enzymes such as QCR1 (cytochrome b-c1 complex subunit 1, mitochondrial), ALDR (aldo-keto reductase family1 member B1), and AL3A1 (aldehyde dehydrogenase, dimeric NADP-preferring) related to carbohydrate turnover, which support the growth of cancer cells and reinforce their aggressiveness, or others like GSHB (glutathione synthetase) related to drug resistance [81]. Differently, exosome proteins were mainly related to cell–matrix adhesion (procollagen III), cell migration/aggressiveness (moesin, S100-A14 protein), and chemotherapy resistance (DNA-directed RNA polymerase II, subunit RPB11-a). Interestingly, the expression of certain further proteins in EVs was significantly increased when the cultured GSCs, the EV source in our experimental model, were exposed to the stimulation of P2X7 receptors [82], which have been recognized as involved in tumor malignancy [83]. Indeed, P2X7 receptor stimulation upregulated the expression in EVs of cytoskeletal proteins involved in cell migration, antioxidant and proteasomal enzymes related to poor survival of glioma patients, and proteins with a regulatory function on chromatin remodeling and transcription.

Around the same period, another Italian group purified small EVs from U87, U373, and GSC cell culture media and analyzed them for proteome, and their research also extended to the expression profile of several microRNAs (miRNAs), which could be able to control gene expression and transcription in recipient cells [84]. As for the proteome evaluation, EVs from all cells shared 788 proteins in their content, most of which had been reported in previous proteomic studies [74,77] and are reported in Vesiclepedia 4.1 (www.microvesicles.org, released on 15 August 2018), according to the suggestion of the same authors. Among the great number of isolated proteins in the EVs obtained from the cell samples, the authors assessed the presence of 36 proteins, including transmembrane proteins and tetraspanins (CD81, CD9, G-proteins, integrins, Ras-related proteins, transferrin receptor protein, basigin), as well as cytosolic proteins (tumor susceptibility gene 101 protein, flotilin-1, Annexins, Alix, Syntenin-1, chaperones). However, there were also differences in the protein cargo between EVs isolated from the medium of GBM cell lines and GSCs. Interestingly, the latter overexpressed some proteins, as we found in our GSC culture medium, namely annexins, heat shock proteins, moesin, and copine [79,82]. These proteins, as well as the many others isolated from the samples, are involved in the modulation of molecular pathways, which could help the recipient cells to react to external stimuli.

### 3.3. Proteomics Applied to EVs Obtained from Patients’ Biological Fluids

Biological fluids such as blood and CSF from GBM patients have been investigated to evaluate the presence of proteins that could be used as tumor biomarkers [85,86,87,88,89]. Although CSF has the advantage of exclusively flowing close to the healthy or tumoral brain parenchyma and, therefore, may represent a better source than plasma to isolate EVs from gliomas and investigate their content [90], it has only recently been used for this aim, often in comparison with blood, in GBM animal models [91] and patients [92,93,94,95]. Thus, RNAs were initially obtained from them, providing useful information for early glioma diagnosis and thus avoiding tumor biopsy [96,97]. The late use of these fluids for EV proteomic studies is likely due to the evidence that both of them, mainly blood, contain a very large number of contaminant proteins that need to be removed to assure the purity of the EV pool, as well as the correct proteome evaluation. Accordingly, procedures have been suggested to isolate purified EVs from these fluids [98].

In one of these studies, blood samples were collected from a glioma patient cohort of 136 patients prior to and 3 weeks post-surgery but before any therapeutic treatment. EVs were isolated by size-exclusion chromatography, and characterized by nanoparticle tracking analysis and electron microscopy, while EV proteome was quantified by multiplex proximity extension immunoassay and analyzed by LC-MS/MS. Procedures for EV isolation from plasma were carefully validated, and data on plasma EVs were compared with those obtained from EVs isolated from cultured glioma cell lines (U87MG). Among the plasma-derived EV proteins, the authors identified syndecan 1 (SDC1) as a valid biomarker able to discriminate between GBMs and low-grade glioma. These findings induced the authors to claim that blood EVs can be considered a promising tool for monitoring gliomas and improving their management [99]. Likewise, plasma was collected from healthy volunteers and patients with GBM or other nervous system tumors, in this case, before and 3 days after surgery. EVs were isolated by ultracentrifugation and adequately characterized (NTA, TEM, and EV concentration; WB analysis for protein markers), while transported proteins were analyzed by MS. This analysis revealed specific protein signatures in GBM-EVs, identifying proteins involved in exosome biogenesis and function, such as ribosomal proteins, annexins, integrins, heat shock proteins, G proteins, Ras-related proteins, tetraspanins, and histones. Furthermore, by analyzing the proteins expressed in EVs from three different GBMs, 11 common proteins, vWF (von Willebrand factor), amyloid P component, serum (APCS), C4B (C4b-binding protein), alpha-1-microglobulin (AMBP), apolipoprotein D (APOD), alpha-2-glycoprotein 1, zinc binding (AZGP1), complement component 4 binding protein beta (C4BPB), Serpin3, ferritin light chain (FTL), complement component 3 (C3), and apoliprotein 3 (APOE), involved in complement and coagulation cascade and iron metabolism were identified. These proteins, which were regarded as a “GBM EV protein signature” disappeared after GBM surgery and also allowed distinguishing GBM patients from healthy controls. The selective expression of these biomarkers was then validated by confirming their expression in a large cohort of GBM patients included in the Cancer Genome Atlas GBM dataset (http://cancergenome.nih.gov/, accessed on 2 September 2024) as reported by the same Authors [92]. In another recent article, EVs were isolated from the plasma of pre-operative glioma patients (grade II-IV) and controls. Their proteins were sequenced by SWATH-MS (sequential window acquisition of all theoretical fragment ion spectra mass spectrometry) and data were analyzed using a comprehensive custom-protein library. The profile of the identified proteins correlated with the glioma grade and malignancy [100]. In this case, the authors concluded that plasma-EV proteins analyzed by the proposed proteomic method could be a convenient tool for assessing panels of GBM biomarkers useful as clinical indicators of tumor growth, and response to therapy and recurrence. Another group recently isolated EVs by differential ultracentrifugation from the plasma of GBM patients and volunteers. After the appropriate EV characterization, MS analysis revealed the distinctive expression of inflammatory proteins, for some of which mRNA data were obtained indicating their belonging to the family of the complement and the coagulation, such as vWF, FCGBP (Fc gamma binding protein), C3, Protein S (PROS1), and serpin family E member 1 (SERPINE1), in the nanoparticles from GBM patients. This study confirmed the possibility of using blood as a noninvasive tool for better GBM management [101]. 

Saliva is now receiving great attention, since it is a less complex biological mixture than plasma and its collection is non-invasive, easy, and inexpensive [102]. It is also possible to isolate EVs from this biological fluid [103] and use them for proteome analysis. Thus, very recently, small EVs were isolated for the first time from the saliva of GBM patients, while EVs from this fluid had previously been investigated in other cancers (for example, [104,105]). Saliva was collected from patients before and following GBM surgery. After isolation, EVs were characterized by cell morphology and known cell-surface protein markers, while their protein content was examined by MS. Although significant differences were not revealed in the size and concentration of EVs derived from saliva collected from pre- and post-surgical GBM patients, a higher protein number was determined in the preoperative samples. Of the latter, those collected from patients with poor prognosis showed a higher expression of four proteins, namely aldolase A, 14-3-3 protein ε, enoyl CoA hydratase 1, and transmembrane protease serine 11B. All these proteins play a role in cell proliferation and migration in gliomas, as well as in other tumors [106]. Furthermore, functional analyses using known databases like gene ontology (GO) and Kyoto Encyclopedia of Genes and Genomes (KEGG) pathways pointed out that many proteins identified in saliva samples from GBM patients may participate in fundamental biological processes such as immune response, catabolic processes involving proteasome, modulation of the cell cycle, and apoptosis. The authors of the article concluded that their findings suggested that salivary EVs could be a good and noninvasive tool to investigate prognostic GBM biomarkers, even though validation studies are necessary.

In addition to saliva, urine is another fluid from which EVs can be isolated, with the urinary system being the principal route of EV clearance. This fluid was very recently used for detecting GBM-associated biomarkers [107]. Urine samples were collected from 24 catheterized GBM patients at different time points, namely immediately before (n = 17) and after (n = 9) surgical removal of primary (n = 17) tumors and before surgery on recurrent tumors (n = 7). The results from these samples were compared with those obtained from healthy controls matched for age and gender. EVs isolated by differential ultracentrifugation were characterized, and the extracted proteins were analyzed by liquid chromatography tandem mass spectrometry coupled to DIA (DIA-LC-MS/MS). Among the great number (6857) of identified proteins, 903 proteins were found in most EVs (>80%) from all sample cohorts, including 94 EV marker proteins which were listed among the first 100 proteins published in a recent update of Vesiclepedia (www.microvesicles.org), as indicated by the Authors of the article. Among these proteins, some specific for GBM were determined as putative urinary–EV biomarkers corresponding to tumor growth and recurrence. In this case, the authors, besides emphasizing that urine is a viable source of GBM biomarkers, suggested that the entire procedure and the biomarker panels detected deserve further investigation.

### 3.4. Proteomics Applied to EVs Obtained from Brain Tumors

As reported above, most studies have been performed using cell culture media and biological fluids from GBM patients, which are more easily accessible as an EV source. However, these nanoparticles may not properly represent the specificity of those directly isolated from the TME. Only recently, EVs were obtained directly from brain tumors, i.e., surgical biopsies of GBM and meningiomas, to study their protein content. EVs were isolated by a series of centrifugation followed by UC, and characterized by NTA and TEM. Through proteomic analysis, the authors, while confirming the presence of numerous proteins previously found in EVs from different sources (for example, in [74,75]), identified novel proteins, which included those associated with solute carriers and fatty acid transport. Of interest, both of them contribute to GBM progression, the former by the activation of the PI3 kinase/AKT molecular pathways and the latter by increasing ATP generation from the oxidation of fatty acids. Finally, some proteins detected in tumor-derived EVs were present only in these nanoparticles and not in the corresponding tumors, and are involved in EV biogenesis, including transmembrane proteins such as CD63, synthenin-1 (SDCBP), vesicle-associated membrane protein 2 (VAMP2), and CD44A [108].

Thus, these results, in our opinion, underline the need to evaluate, more extensively and in depth, the protein content of EVs obtained directly from solid tumors and to compare these results with those obtained so far from EVs from liquid sources.

## 4. Discussion and Concluding Remarks

As can be inferred from the data reported above, there has been a very large number of proteins identified in EVs from cell line cultures, which in part are similar to those identified in EVs obtained from GBM patients’ cells and fluids. They are mostly membrane proteins (i.e., integrins or annexins), as well as intracellular proteins, including structural proteins (actins, vimentin), chaperones, mitochondrial, antioxidants, and proteasomal enzymes. Although most of these proteins are also dysregulated in GBM tissues, their role in tumor progression has not been well characterized, nor their relationship with the molecular mechanisms underlying GBM malignancy. To date, some reports have indicated that GBM-derived exosomes, through their cargo of RNA and/or proteins, can enhance GSC stemness, inhibiting their differentiation, while GBM-derived microvesicles can stimulate angiogenesis by acting on endothelial cell proliferation [109]. Again, proteins transported in GBM-derived EVs can suppress the monocyte immune activity, favoring tumor expansion [110]. Nevertheless, information on EV protein content is still scattered, as is their link with signal pathways supporting GBM progression. Hope in this direction was given by a recent article that demonstrated that EVs, mostly exosomes, may act on GSCs and nontumor stromal cells upregulating the mTOR pathway, which in turn depresses autophagy, thus promoting GSC stemness and expansion, as well as exosome release [111].

Accordingly, it is not surprising that there have been no useful protein biomarkers, univocally selected from EVs, which can be used to accelerate GBM diagnosis or to open a novel therapeutic avenue for GBM management. Hopefully, this impasse will lead to improving the quality and efficiency of research on the EV proteome and reflecting on what are the best ways to make this research more fruitful, so that researchers, and especially GBM patients, benefit from the resulting findings. 

To achieve this, various aspects need to be examined. For instance, one critical aspect concerns the procedures related to (i) isolation of a purified EV pool; (ii) EV characterization; as well as the (iii) lysis and (iv) extraction of the proteins. Although the last ISEV article [MISEV 2024] provided a series of the minimal information required for studies on EV content, there is no procedure yet recognized as the “gold” standard for the steps indicated above [112].

In relation to the points mentioned above, it should be underlined that while the use of conditioned media from glioma cell lines leads to obtaining EV pools exclusively derived from those cancer cells, EV isolation from patient biological fluids is challenging [113]. Indeed, in addition to the relatively low abundance of brain-derived EVs in those fluids, due to the huge amount of different EVs derived from peripheral tissues, the lack of specific surface neural markers assuring their cell origin is crucial [114]. 

With regard to studies on the proteome of GBM-derived EVs, in most of them, there was a certain variability concerning the experimental design, sample collection, and processing, as well as in the data collection. For greater usefulness of the data so obtained, it would be desirable to harmonize the aforementioned steps. In particular, EVs should be collected from GBMs and healthy patients to compare their proteome by MS analysis. Furthermore, GBM-EV proteins with a statistically significant increase or decrease, the list of which could be very large compared to those from control-EVs, should be validated by further assays. For example, it would be necessary to (i) select certain proteins based on their higher/lower expression in the examined tumor samples; (ii) confirm their identity and increase/decrease by immunoassays (ELISA and/or WB analyses); (iii) evaluate the expression of genes related to the selected proteins and perform immunohistochemistry on tissues from a distinct cohort of patients; and (iv) perform all statistical tests, i.e., receiver operating characteristic (ROC) curve analysis, to confirm the candidature of the selected proteins as potential tumor biomarkers [115]. Moreover, the use of more recent technologies should be encouraged, such as those using TIMS coupled to TOF mass analysis, as well as advanced MS data acquisition by PASEF. This proteomic procedure was recently used to analyze the proteome of nononcologic patient plasma and EVs isolated from these samples, revealing that over 90% of the proteins were identified in neat plasma and EV fractions. However, only some of them were enriched in the EVs, thus confirming that many blood proteins are strongly, although non-specifically, bound to nanoparticles [116].

It is also worth highlighting that complex procedures, mainly based on the “bottom-up” or “shotgun” proteomics, have led to the discovery of highly abundant amino acid canonical sequences, which, however, are often inferred on a pair of peptides [67]. This kind of massive proteomics has so far been revealed to be ineffective and clinical translation remains elusive. Probably, efforts should be focused on different approaches, as recently pointed out. 

For example, some researchers have emphasized the need to provide deep proteomic analyses addressing proteo-forms, that is proteins present as different isoforms due to post-translational modifications (PTMs) in native samples/systems and which do not directly correspond to genes. To achieve this, there is the possibility of studying post-translational modification of certain proteins utilizing the immunoaffinity enrichment of proteins, selected for the presence of certain amino acid residues that can undergo modification, followed by high-resolution mass spectrometry [117]. Alternatively, “top-down” proteomics can be used, which allows protein identification by either TIMS, to store an isolated protein ion for mass measurement and tandem mass spectrometry (MS/MS) analysis, or other protein purification methods such as two-dimensional gel electrophoresis in conjunction with MS/MS. Challenges are also found in the adoption of this method and include, among others, protein solubility, proteome complexity, intact protein data analysis, and proteoform–function relationships [118,119]. Of note, there is now the possibility of investigating the structure of large protein complexes maintaining biomolecules in their natural folded state by a new type of MS called “native” MS analysis. This is possible with new instruments recently developed by two companies, namely Thermo-Fisher Scientific and Bruker, which rely on high-resolution MS and allow protein identification, without submitting them to enzymatic digestion. This strategy could lead to measuring exact protein mass with more sophisticated techniques, for top-down analysis of native protein complexes [120].

The top-down approach can also be applied to find protein domain mutations that significantly affect cancer onset and growth by altering protein structure, function, and signals [121]. In this case, top-down proteomics, which can analyze intact proteins, should be used, instead of the traditional “bottom-up” proteomics, whose procedure implies protein digestion into peptides. When identified, protein domain mutations can provide useful information about the severity of the disease and the patient’s response to treatment, while predicting possible resistance to targeted therapy [122,123]. This aspect assumes particular relevance in GBM, where mutations are multiple and sustain tumor progression. Hence, EVs, which reflect the tumor state, could offer the most suitable tool to explore this aspect. Finally, we cannot forget other substantial features. One of these is the need to also examine the protein content of EVs directly obtained from GBM specimens. This investigation should help in finding new biomarkers that more specifically indicate the type and grade of the related tumor. In addition, it would be important to evaluate how many and which EVs are taken up by tumor and non-tumor cells, and the consequences of such uptake. 

**Figure 3:**
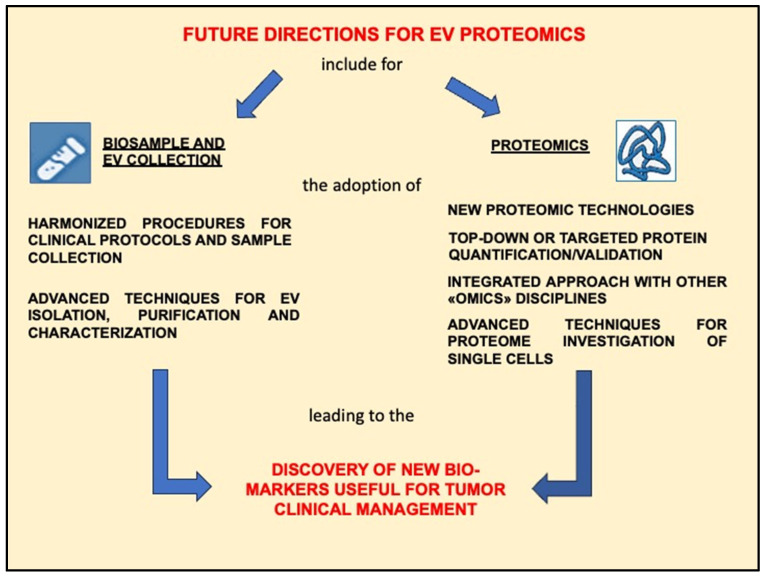
Challenges to be addressed in order to be able to use proteomic data from the study of EV proteins in a useful way for the treatment of patients with GBM.

In conclusion, EV proteome analysis is still in its early stages, and the problems to be solved are many and involve not only EV biology and functions, but also the significance of proteomic analyses, for progress in understanding tumors. Nevertheless, research will surely progress in this field, offering new analytical devices/instruments to improve proteome analysis and knowledge, as well as computerized systems and databases to speed up protein identification and related functions (Figure 3).

Indeed, proteomics has so far identified a great number of proteins in tumors and EVs derived from them. The complexity of these data requires ever more sophisticated approaches to develop biomarker panels useful for tumor diagnosis and management. Therefore, new methods based on the use of the artificial intelligence (AI) are now being developed to increase the efficiency of research in oncology [124]. This aspect has also been applied to EV proteomics. Thus, EV characterization based on microfluidics has been associated with AI, allowing the characterization of single EVs, which could improve the application of EVs for precision medicine [125]. Moreover, AI can also be applied to improve the identification and study of a larger number of EVs. Thus, a branch of AI, namely machine learning (ML), relying on algorithms to analyze input data and to make predictions from them with significant accuracy, has been integrated with advanced microfluidic techniques such as surface-enhanced Raman scattering (SERS). This pairing can enhance the Raman scattering by molecules adsorbed on rough metal surfaces or other nanostructures, potentiating detection up to single molecules. This strategy could allow detecting EVs, particularly sEVs, giving an opportunity to better analyze their content [126]. In addition, ML could be applied to data deriving from high-resolution imaging obtained through the use of mass spectral imaging (MSI) techniques for EV analysis. For that aim, time of flight secondary ion mass spectrometry (TOF-SIMS) was employed, which allowed collecting high-resolution and spatial information on EVs [127]

Of course, we should not forget to mention that a deeper picture of tumor malignancy, as well as the identification of putative druggable targets, will be achieved only by integrating proteomic data with data coming from the other “omics” sciences [128]. This last but essential passage should allow a better understanding of tumor complexities and finding more successful antitumor therapies. Once again, the help of AI seems to be crucial to achieve this goal [124,128].

## Figures and Tables

**Table 1:** Pros and cons of different EV isolation procedures.

Isolation Procedure	Pros	Cons	References
Differential ultracentrifugation (UC)	Still considered the gold standard for exosome isolation; less expensive than DG-UC	Costly equipment; high volume requirement; possible mechanical damage and incomplete EV isolation from contaminants	[26]
Density gradient ultracentrifugation (DG-UC)	High recovery Elevated EV purity and integrity	Pre-purification of samples; time-consuming procedure	[27]
Ultra-filtration (UF) + size exclusion (SE) chromatography	Suitable for analyzing large volumes. Fast and high recovery efficiency	Requires an appropriate choice of filter pore size and further purification	[28]
EV–polymer interaction/precipitation (A) or immuno- affinity capture by antigen/antibody binding (B)	Quick and easy; (A and B): EV recovery higher than UC.	(A) Low purity, protein contaminants. (B) Expensive, not suitable for large samples and EV sorting by size	[29]
Microfluidic techniques (±optical or spectroscopic devices) using a passive approach by passive filtration through nano-porous membranes (A) active approach exploiting physical forces applied to EV-containing fluids or suitable substrates coupled to antibodies for selected EV antigens (B)	Membranes remove cell debris allowing the sEV passage; high recovery Ability to analyze small volumes; quick and high recovery	Figure 1 Potential damage to EVs due to shear stress A and B still require validation and standardization	[23,30,31,32,33] [34,35]

**Table 2:** Pros and cons in EV characterization procedures.

Characterization Procedure	Pros	Cons	References
Dynamic light scattering (DLS)	Time-scale fluctuation of scattered light determines the diffusion coefficient and hence the size of particles	Capable of determining 1–6 µm particles, not suitable for characterizing EVs with heterogeneous size distribution	[39]
NTA (nanoparticle tracking analysis)	Particle size and concentration with limit detection below 100 nm	Contaminant particles can be included; high-cost tools	[40,41]
TRPS (tunable resistive pulse sensing)	Measurement of particle zeta potential (related to surface charge) and size simultaneously; information on EV concentration for each size population	Frequent instrument calibration	[42]
Flow cytometry	High throughput measurements, evaluation and quantification of the surface protein	Limitation in EV size analysis due to detection of coincident events or swarm effects	[43,44]
Micro-bicinchoninic acid (BCA) method	Total protein concentration measurement (colorimetric assay)		[41,45,46]
ELISA assay	Detection of specific antigens (i.e., tetraspanins: CD63, CD9, CD81) as EV biomarkers	Limitation in EV size analysis due to detection of coincident events or swarm effects	[37,44]
Electron microscopy (EM)	Among the various EM techniques, transmission electron microscopy (TEM) and cryo-electron microscopy (cryo-EM) have been commonly used for EV image characterization	Longer procedure for TEM than for cryo-TEM sample preparation; high cost of the instrument	[47]
Atomic force microscopy (AFM)	High-resolution images	High cost of the instrument; possible damage to EV morphology during scanning	[46]

Other references in which further information can be found about the procedures for EV characterization are [29,48,49].

## Data Availability

Data reported in this review can be found in the cited references.
